# Monocyte to high‐density lipoprotein ratio predicts clinical outcomes after acute ischemic stroke or transient ischemic attack

**DOI:** 10.1111/cns.14152

**Published:** 2023-03-13

**Authors:** Qin Xu, Qiong Wu, Lu Chen, Hao Li, Xue Tian, Xue Xia, Yijun Zhang, Xiaoli Zhang, Yongzhong Lin, Yiping Wu, Yongjun Wang, Xia Meng, Anxin Wang

**Affiliations:** ^1^ Department of Neurology, Beijing Tiantan Hospital Capital Medical University Beijing China; ^2^ China National Clinical Research Center for Neurological Diseases, Beijing Tiantan Hospital Capital Medical University Beijing China; ^3^ Beijing Municipal Key Laboratory of Clinical Epidemiology Beijing China; ^4^ Department of Neurology The Second Hospital of Dalian Medical University Dalian China; ^5^ Department of Neurology, ZiBo Central Hospital Zibo China; ^6^ Department of Epidemiology and Health Statistics, School of Public Health Capital Medical University Beijing China; ^7^ Department of Neurology, HanDan Central Hospital Handan China; ^8^ Advanced Innovation Center for Human Brain Protection Capital Medical University Beijing China; ^9^ Center for Excellence in Brain Science and Intelligence Technology Chinese Academy of Sciences Shanghai China

**Keywords:** death, functional outcome, high‐density lipoprotein, monocyte count, stroke

## Abstract

**Aims:**

The monocyte to high‐density lipoprotein cholesterol ratio (MHR) has emerged as a novel inflammatory biomarker of atherosclerotic cardiovascular disease. However, it has not yet been identified whether MHR can predict the long‐term prognosis of ischemic stroke. We aimed to investigate the associations of MHR levels with clinical outcomes in patients with ischemic stroke or transient ischemic attack (TIA) at 3 months and 1 year.

**Methods:**

We derived data from the Third China National Stroke Registry (CNSR‐III). Enrolled patients were divided into four groups by quartiles of MHR. Multivariable Cox regression for all‐cause death and stroke recurrence and logistic regression for the poor functional outcome (modified Rankin Scale score 3–6) were used.

**Results:**

Among 13,865 enrolled patients, the median MHR was 0.39 (interquartile range, 0.27–0.53). After adjustment for conventional confounding factors, the MHR level in quartile 4 was associated with an increased risk of all‐cause death (hazard ratio [HR], 1.45; 95% confidence interval [CI], 1.10–1.90), and poor functional outcome (odd ratio [OR], 1.47; 95% CI, 1.22–1.76), but not with stroke recurrence (HR, 1.02; 95% CI, 0.85–1.21) at 1 year follow‐up, compared with MHR level in quartile 1. Similar results were observed for outcomes at 3 months. The addition of MHR to a basic model including conventional factors improved predictive ability for all‐cause death and poor functional outcome validated by the C‐statistic and net reclassification index (all *p* < 0.05).

**Conclusions:**

Elevated MHR can independently predict all‐cause death and poor functional outcome in patients with ischemic stroke or TIA.

## Graphical abstract

Higher monocyte to high‐density lipoprotein ratio was associated with a high risk of all‐cause death and poor functional outcome at 3 months and 1 year in patients with ischemic or TIA. Elevated MHR can independently predict all‐cause death and poor functional outcome in patients with ischemic stroke or TIA.

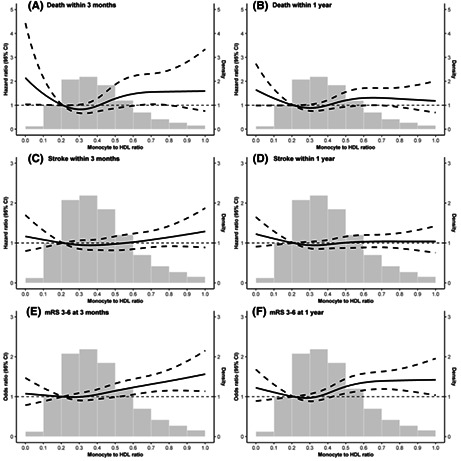

## 1 INTRODUCTION

Atherosclerosis, especially intracranial atherosclerosis, is the key etiological factor of ischemic stroke. Inflammation and lipid accumulation are recognized as fundamental to the pathophysiology and progression of atherosclerosis.^1^, ^2^ Several inflammatory factors have been associated with strokes, such as neutrophil‐to‐lymphocyte ratio,^3^ C‐reactive protein (CRP) or hypersensitive CRP (hsCRP),^4^ and d‐dimer.^5^ Monocytes, as agents of the innate immune response,^6^, ^7^ interact primarily with platelets and endothelial cells to aggravate inflammatory, pro‐thrombotic pathways which in turn cause the formation, progression, and rupture of atherosclerotic plaques.^8^, ^9^ As such, monocytes are emerging as indicators of post‐ischemic stroke inflammation.^10^ Lipid levels, especially the high‐density lipoprotein (HDL) cholesterol, can impact monocyte development. Murine models showed that HDL can suppress stem cell proliferation and then reduce monocytosis.^11^, ^12^ HDL is inversely associated with intermediate monocyte counts^13^, ^14^ and can counteract the pro‐inflammatory and pro‐oxidant effects of monocytes,^15^ thus reducing the risk of atherosclerotic events. Recently, the monocyte count to HDL ratio (MHR), as calculated by dividing the monocyte count by the HDL value, has been recognized as a novel inflammatory biomarker of atherosclerotic cardiovascular diseases,^16^ including ischemic stroke.^17^ It has been reported to be associated with poor outcomes among patients with the acute coronary syndrome,^18^ the recurrence of atrial fibrillation,^19^ stroke‐associated pneumonia in patients with acute ischemic stroke (AIS),^20^ and the risk of disability or death in patients with cerebral hemorrhage.^21^ Nevertheless, to the best of our knowledge, evidence on the role of MHR in the prognosis of ischemic stroke is limited and conflicting. Bolayir et al.^22^ conducted a case–control study including 466 patients with AIS and found that high MHR was associated with an increased risk of 30‐day mortality after stroke. Another observational study involving 803 patients with AIS found no association between MHR and 3 months mortality.^20^ In addition, it has not yet been identified whether MHR can predict recurrent stroke, poor functional outcome, and long‐term mortality after ischemic stroke using prospective cohort data.

Therefore, this study aimed to investigate the associations between MHR and clinical outcomes among patients with ischemic stroke or transient ischemic attack (TIA) based on data from the Third China National Stroke Registry (CNSR‐III).

## 2 METHODS

### 2.1 Study design and population

CNSR‐III is a large‐scale, nationwide, hospital‐based, prospective registry for patients with ischemic stroke or TIA who presented to hospitals between August 2015 and March 2018 in China. The registry consecutively enrolled 15,166 patients from 201 hospitals in 22 provinces and 4 municipalities. The inclusion criteria were (1) aged ≥18 years old; (2) ischemic stroke or TIA within 7 days from the onset of symptoms to enrollment; and (3) informed consent from the patients or legally authorized representatives. Details of the design and procedure of the CNSR‐III have been published elsewhere.^23^ The study was approved by the ethics committee of Beijing Tiantan Hospital and participant hospitals. Written informed consent was obtained from the patients or their legally authorized representatives.

### 2.2 Data collection and calculation

Baseline data on demographics and clinical characteristics, including age, sex, body mass index, smoking and alcohol consumption status, diseases history (hypertension, stroke or TIA, diabetes, dyslipidemia, atrial fibrillation, coronary heart disease, heart failure, peripheral vascular disease, and arthritis), prestroke modified Rankin Score (mRS) score, the National Institutes of Health Stroke Scale (NIHSS) score at admission, and medications provided in hospital care (antihypertensive agents, antiplatelet agents, anticoagulant agents, cholesterol‐lowering agents, and hypoglycemic agents), were collected by trained research coordinators at each center via face‐to‐face interviews or medical records. The etiological classification of the index events was performed by the Trial of Org 10172 in Acute Stroke Treatment (TOAST) criteria.^24^

Fasting whole blood samples from venipuncture were collected in vacutainer tubes containing EDTA within 24 h of admission. Afterward, the monocyte count was tested by an automated hematology analyzer at each research center. The blood samples were frozen in a cryotube at −80°C refrigerator and were transported to the central laboratory in Beijing Tiantan Hospital by cold chain. Serum total cholesterol (TC), HDL, low‐density lipoprotein (LDL), and triglyceride (TG) were analyzed centrally. All laboratory indicators were performed by laboratory personnel who were unaware of patients' clinical characteristics. MHR was calculated as the ratio of blood monocyte count to HDL concentration.

### 2.3 Outcome assessment

Patients were followed up by trained research coordinators who were blinded to subjects' baseline characteristics at 3 months and 1 year after symptom onset. Data regarding all‐cause death, stroke recurrence, and modified Rankin Scale (mRS) score were collected. All‐cause death was defined as death from any cause and confirmed by a death certification from the attended hospital or the local citizen registry. The stroke recurrence was defined as a new ischemic stroke or hemorrhagic stroke within 3 months and 1 year after symptom onset. Poor functional outcome was defined as mRS score ranging from 3 to 6 or from 2 to 6 at 3 months and 1 year. The definitions of the above outcomes were consistent with those previously published in the CNSR‐III protocol.^23^

### 2.4 Statistical analysis

Participants were classified into four groups by MHR quartiles as follows: Q1 (<0.27), Q2 (0.27–0.39), Q3 (0.39–0.53), and Q4 (≥0.53), respectively. The normality of the data distribution was evaluated by the Kolmogorov–Smirnov method. Continuous variables were expressed as medians with interquartile ranges (IQRs) due to nonnormally distributed and categorical variables as frequencies and percentages. Baseline data were compared across MHR quartile groups using the Kruskal–Wallis test for continuous variables, and the chi‐square test or Fisher's exact test for categorical variables. The Kaplan–Meier method and the log‐rank test were used for time‐to‐event data. The associations of MHR with all‐cause death and stroke recurrence were explored by Cox proportion hazards models, and hazard ratios (HRs) and 95% confidence intervals (CIs) were reported. The proportionality assumption was assessed by scaled Schoenfeld residuals, and there was no violation of the assumptions. For poor functional outcomes, odds ratio (OR) with 95% CI was estimated by the logistic regression model. A robust sandwich variance estimator was used to deal with the correlations for clustering by hospital. We fitted an unadjusted model with no confounding factors and three adjusted models. Variables adjusted in model 1 included age and sex, and in model 2 further included body mass index, current smoker, current alcohol drinking, disease history (hypertension, stroke or TIAs, diabetes, dyslipidemia, atrial fibrillation, arthritis), NIHSS score at admission, stroke subtype, prestroke mRS score, stroke etiology, antihypertensive agents, cholesterol‐lowering agents, and hypoglycemic agents. Model 3 was additionally adjusted for TC, TG, and LDL. A linear trend test was performed by treating the median MHR value of each quartile as a continuous variable in each model. Moreover, we used a restricted cubic spline with adjustment for potential covariates to evaluate the patterns of relationships between continuous MHR value and clinical outcomes. Additionally, *C* statistics, integrated discrimination improvement (IDI), and net reclassification index (NRI) were calculated to establish the predictive performance of MHR added to the basic model. Subgroup analyses were performed according to age, sex, BMI, NIHSS score, history of stroke or TIA, and stroke etiology with an interaction test. All analyses were performed using SAS software version 9.4 (SAS Institute Inc.), and R version 4.0.2. Overall, a two‐sided *p*‐value of <0.05 was assumed statistically significant.

## 3 RESULTS

### 3.1 Baseline characteristics

Among the enrolled patients in the CNSR‐III, we excluded patients without baseline monocyte (*n* = 293), HDL (*n* = 601), or who were lost to follow‐up (*n* = 407), and accordingly, a total of 13,865 patients were included in our analyses. Table S1 showed that baseline characteristics of included and excluded patients were largely comparable, except that the included patients were more likely to be younger, females, non‐smokers, had lower NIHSS scores at admission, and received a higher proportion of TIA and antiplatelet agents but a lower proportion of anticoagulants and rt‐PA intravenous thrombolytic.

The baseline characteristics of our study population stratified according to the MHR quartile are shown in Table 1. Compared to patients with a lower MHR, those in the highest quartile group were more likely to be younger, males, current smokers, alcohol drinkers, having higher BMI and prestroke mRS score, higher prevalence of medical history (including hypertension, diabetes, dyslipidemia, and atrial fibrillation) and large‐artery atherosclerosis ischemic stroke, higher TG concentration, and higher proportion of antihypertensive agents and hypoglycemic agents, but lower arthritis, TC concentration, and LDL concentration.

**TABLE 1:** Baseline characteristics according to quartiles of MHR.

Characteristics	Total	Quartiles of MHR				*p* Value
		Quartile 1	Quartile 2	Quartile 3	Quartile 4	
		<0.27	0.27–0.39	0.39–0.53	≥0.53	
No. of the patients	13,865	3371	3610	3353	3531	
Age, median (IQR), years	62 (54–70)	63 (56–70)	63 (55–70)	63 (54–70)	62 (53–70)	<0.001
Women, *n* (%)	4435 (31.99)	1629 (48.32)	1255 (34.76)	916 (27.32)	635 (17.98)	<0.001
BMI, median (IQR), kg/m^2^	24.49 (22.6–26.56)	24.14 (22.04–26.04)	24.49 (22.6–26.57)	24.49 (22.85–26.57)	24.8 (22.99–26.83)	<0.001
Current smoker, *n* (%)	4302 (31.03)	745 (22.10)	1032 (28.59)	1122 (33.46)	1403 (39.73)	<0.001
Current alcohol drinking, *n* (%)	6214 (44.82)	1274 (37.79)	1569 (43.46)	1581 (47.15)	1790 (50.69)	<0.001
Medical history, *n* (%)						
Hypertension	8666 (62.50)	2055 (60.96)	2253 (62.41)	2083 (62.12)	2275 (64.43)	0.026
Stroke or TIA	3376 (24.35)	780 (23.14)	884 (24.49)	799 (23.83)	913 (25.86)	0.056
Diabetes	3223 (23.25)	676 (20.05)	772 (21.39)	845 (25.20)	930 (26.34)	<0.001
Dyslipidemia	1096 (7.90)	211 (6.26)	276 (7.65)	296 (8.83)	313 (8.86)	<0.001
Atrial fibrillation	924 (6.66)	201 (5.96)	227 (6.29)	225 (6.71)	271 (7.67)	0.026
Coronary heart disease	1477 (10.65)	353 (10.47)	367 (10.17)	377 (11.24)	380 (10.76)	0.515
Heart failure	84 (0.61)	19 (0.56)	20 (0.55)	25 (0.75)	20 (0.57)	0.696
Peripheral vascular disease	111 (0.80)	25 (0.74)	25 (0.69)	29 (0.86)	32 (0.91)	0.717
Arthritis	303 (2.19)	93 (2.76)	83 (2.30)	65 (1.94)	62 (1.76)	0.024
Admission stroke data						
NIHSS at admission, median (IQR)	3 (1–6)	3 (1–6)	3 (1–6)	3 (1–6)	3 (1–6)	<0.001
Prestroke mRS score 2–5, *n* (%)	1227 (8.85)	266 (7.89)	299 (8.28)	311 (9.28)	351 (9.94)	0.011
Stroke subtype, *n* (%)						<0.001
Ischemic stroke	12,908 (93.10)	3099 (91.93)	3343 (92.60)	3122 (93.11)	3344 (94.70)	
TIA	957 (6.90)	272 (8.07)	267 (7.40)	231 (6.89)	187 (5.30)	
Stroke etiology, *n* (%)						<0.001
Large‐artery atherosclerosis	3492 (25.19)	753 (22.34)	895 (24.79)	877 (26.16)	967 (27.39)	
Cardioembolism	844 (6.09)	198 (5.87)	211 (5.84)	188 (5.61)	247 (7.00)	
Small‐vessel occlusion	2916 (21.03)	764 (22.66)	789 (21.86)	724 (21.59)	639 (18.10)	
Other determined etiology	167 (1.20)	38 (1.13)	47 (1.30)	31 (0.92)	51 (1.44)	
Undetermined etiology	6446 (46.49)	1618 (48.00)	1668 (46.20)	1533 (45.72)	1627 (46.08)	
Laboratory data, median (IQR)						
TC, mmol/L	4.14 (3.44–4.91)	4.36 (3.68–5.18)	4.22 (3.49–4.93)	4.10 (3.42–4.86)	3.91 (3.25–4.64)	<0.001
TG, mmol/L	1.37 (1.02–1.92)	1.24 (0.93–1.69)	1.37 (1.01–1.86)	1.42 (1.07–1.99)	1.48 (1.12–2.09)	<0.001
LDL, mmol/L	2.45 (1.85–3.11)	2.57 (1.94–3.26)	2.51 (1.90–3.15)	2.43 (1.84–3.07)	2.30 (1.73–2.96)	<0.001
HDL, mmol/L	1.09 (0.91–1.30)	1.33 (1.14–1.56)	1.14 (1.00–1.32)	1.03 (0.90–1.19)	0.89 (0.77–1.03)	<0.001
Monocyte, 10^9^/L	0.42 (0.32–0.54)	0.27 (0.21–0.32)	0.38 (0.32–0.43)	0.47 (0.40–0.54)	0.61 (0.52–0.74)	<0.001
MHR	0.39 (0.27–0.53)	0.21 (0.17–0.24)	0.33 (0.30–0.36)	0.45 (0.42–0.49)	0.67 (0.58–0.81)	–
Treatment in hospital, *n* (%)						
Antihypertensive agents	6384 (46.35)	1479 (44.08)	1652 (45.98)	1561 (46.95)	1692 (48.34)	0.004
Antiplatelet agents	13,387 (97.20)	3266 (97.35)	3491 (97.16)	3233 (97.23)	3397 (97.06)	0.905
Anticoagulant agents	1391 (10.10)	337 (10.04)	358 (9.96)	316 (9.50)	380 (10.86)	0.308
Cholesterol‐lowering agents	13,270 (96.35)	3232 (96.33)	3474 (96.69)	3192 (96.00)	3372 (96.34)	0.507
Hypoglycemic agents	3477 (25.25)	705 (21.01)	852 (23.71)	912 (27.43)	1008 (28.80)	<0.001
rt‐PA intravenous thrombolytic	1168 (8.42)	281 (8.34)	311 (8.61)	284 (8.47)	292 (8.27)	0.956
Mechanical thrombectomy	39 (0.28)	10 (0.30)	9 (0.25)	11 (0.33)	9 (0.25)	0.917

Abbreviations: BMI, body mass index; HDL, high‐density lipoprotein; IQR, interquartile range; LDL, low‐density lipoprotein; MHR, monocyte‐to‐HDL ratio; mRS, modified Rankin Scale; NIHSS, the National Institutes of Health Stroke Scale; rt‐PA, recombinant tissue plasminogen activator; TC, total cholesterol; TG, triglyceride; TIA, transient ischemic attack.

### 3.2 MHR with clinical outcomes

**FIGURE 1:**
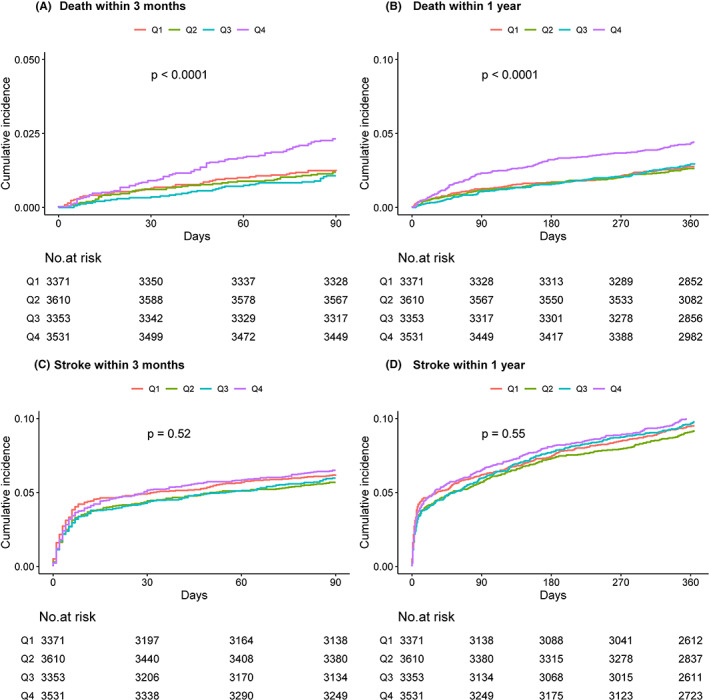
Kaplan–Meier curves for clinical outcomes. (A) Kaplan–Meier curve for all‐cause death within 3 months. (B) Kaplan–Meier curve for all‐cause death within 1 year. (C) Kaplan–Meier curve for stroke recurrence within 3 months. (D) Kaplan–Meier curve for stroke recurrence within 1 year.

**FIGURE 2:**
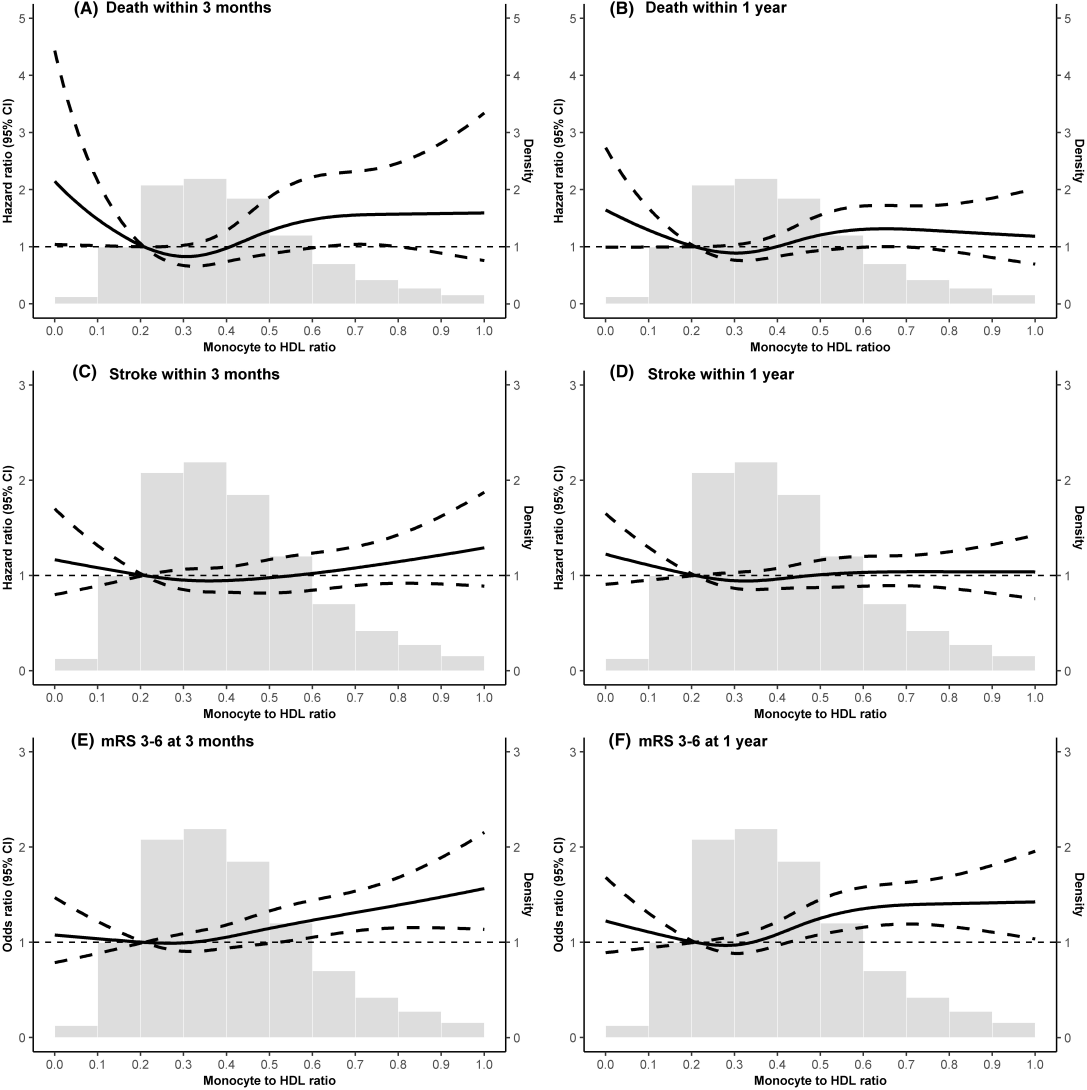
Association between monocyte to HDL ratio and clinical outcomes. Adjusted for age, sex, body mass index, current smoker, current alcohol drinking, disease history (hypertension, stroke or transient ischemic attacks, diabetes, dyslipidemia, atrial fibrillation, and arthritis), the National Institutes of Health Stroke Scale score at admission, stroke subtype, prestroke modified Rankin Scale score, stroke etiology, antihypertensive agents, cholesterol‐lowering agents, hypoglycemic agents, total cholesterol, triglyceride, and low‐density lipoprotein.

Of the 13,865 patients, 2.76% (93) died and 11.09% (374) had a poor functional outcome (mRS 3–6) during 1‐year follow‐up. In addition, 9.43% (318) of patients experienced a recurrent stroke. Figure 1 depicts the cumulative incidence of all‐cause death and stroke recurrence by MHR quartiles. The risks of all‐cause death within 3 months and 1 year were higher in patients with higher MHR levels (log‐rank *p* < 0.001). Table 2 demonstrates the associations of MHR with clinical outcomes. The MHR as a continuous variable was positively associated with all‐cause death (crude HR per 1 standard deviation [SD], 1.09; 95% CI, 1.06–1.12) and poor functional outcome (crude OR per 1 SD, 1.12; 95% CI, 1.03–1.21) at 1 year. The associations became marginally significant in model 3 (adjusted HR per 1 SD, 1.04; 95% CI, 1.00–1.09; *p* = 0.064; adjusted OR per 1 SD, 1.08; 95% CI, 1.00–1.17; *p* = 0.063). When MHR was classified into quartiles, the MHR level in quartile 4 was associated with an increased risk of all‐cause death within 1 year (crude HR, 1.61; 95% CI, 1.25–2.06) compared with quartile 1 in the unadjusted model. After further adjustment for the potential confounders, this association attenuated but remained significant (adjusted HR, 1.45; 95% CI, 1.10–1.89) in model 3. MHR level in quartile 4 was also associated with an increased risk of a poor functional outcome at 1 year (crude OR, 1.56; 95% CI, 1.33–1.83) compared with quartile 1 in the unadjusted model. The association persisted after further adjustment for the potential confounders in model 3 (adjusted OR, 1.47; 95% CI, 1.22–1.76). Similar results were observed when the poor functional outcome was defined as mRS of 2–6 (Table S2). We observed a slightly higher incidence of stroke recurrence within 1 year in patients with the highest quartile of MHR level compared with those in quartile 1 (10.03% vs. 9.43%), however, increased but no significant risk was found in the unadjusted model (crude HR, 1.07; 95% CI, 0.90–1.27) and in model 3 (adjusted HR, 1.02; 95% CI, 0.85–1.21). Similar results were found for outcomes at 3 months. Multivariable‐adjusted restricted spline analysis showed J‐shaped associations of MHR with the risk of poor functional outcome (mRS score 3–6) (Figure 2E,F).

**TABLE 2:** Associations of MHR with all‐cause death, stroke recurrence, and poor functional outcome.

Outcomes	Quartiles of MHR					Per 1 SD increase
	Q1	Q2	Q3	Q4	*p* For trend	
At 3 months						
Death						
*n* (%)	43 (1.28)	43 (1.19)	36 (1.07)	82 (2.32)		
Unadjusted	Reference	0.93 (0.58–1.49)	0.84 (0.55–1.29)	1.83 (1.27–2.62)	<0.001	1.10 (1.08–1.13)
Model 1	Reference	0.98 (0.61–1.57)	0.89 (0.58–1.38)	2.09 (1.42–3.09)	<0.001	1.09 (1.07–1.12)
Model 2	Reference	0.92 (0.56–1.51)	0.86 (0.55–1.35)	1.69 (1.13–2.53)	0.005	1.05 (1.00–1.11)
Model 3	Reference	0.93 (0.56–1.54)	0.88 (0.56–1.39)	1.75 (1.14–2.67)	0.004	1.05 (1.00–1.11)
Stroke recurrence						
*n* (%)	209 (6.20)	205 (5.68)	200 (5.96)	229 (6.49)		
Unadjusted	Reference	0.91 (0.75–1.11)	0.96 (0.78–1.17)	1.05 (0.85–1.29)	0.586	1.01 (0.96–1.06)
Model 1	Reference	0.93 (0.77–1.13)	0.99 (0.81–1.21)	1.10 (0.89–1.37)	0.321	1.02 (0.97–1.06)
Model 2	Reference	0.90 (0.74–1.10)	0.94 (0.77–1.15)	0.99 (0.80–1.23)	0.935	0.99 (0.93–1.05)
Model 3	Reference	0.89 (0.73–1.08)	0.93 (0.76–1.14)	0.98 (0.79–1.22)	0.988	0.99 (0.93–1.05)
mRS score 3–6						
*n* (%)	425 (12.61)	459 (12.71)	430 (12.82)	566 (16.03)		
Unadjusted	Reference	1.01 (0.86–1.18)	1.02 (0.86–1.21)	1.32 (1.12–1.57)	0.001	1.10 (1.03–1.18)
Model 1	Reference	1.06 (0.90–1.25)	1.10 (0.92–1.31)	1.53 (1.28–1.81)	<0.001	1.14 (1.03–1.26)
Model 2	Reference	0.97 (0.82–1.15)	1.00 (0.84–1.21)	1.21 (0.99–1.47)	0.051	1.07 (1.00–1.14)
Model 3	Reference	0.98 (0.82–1.16)	1.02 (0.85–1.22)	1.24 (1.02–1.51)	0.025	1.07 (1.00–1.14)
At 1 year						
Death						
*n* (%)	93 (2.76)	95 (2.63)	99 (2.95)	155 (4.39)		
Unadjusted	Reference	0.95 (0.71–1.28)	1.07 (0.81–1.42)	1.61 (1.25–2.06)	<0.001	1.09 (1.06–1.12)
Model 1	Reference	0.98 (0.73–1.30)	1.10 (0.84–1.44)	1.76 (1.35–2.28)	<0.001	1.08 (1.05–1.11)
Model 2	Reference	0.92 (0.68–1.25)	1.01 (0.76–1.33)	1.39 (1.07–1.82)	0.005	1.04 (1.00–1.09)
Model 3	Reference	0.94 (0.69–1.27)	1.03 (0.78–1.37)	1.45 (1.10–1.89)	0.003	1.04 (1.00–1.09)
Stroke recurrence						
*n* (%)	318 (9.43)	328 (9.09)	325 (9.69)	354 (10.03)		
Unadjusted	Reference	0.96 (0.82–1.12)	1.02 (0.87–1.20)	1.07 (0.90–1.27)	0.356	1.02 (0.97–1.06)
Model 1	Reference	0.97 (0.83–1.14)	1.05 (0.89–1.23)	1.11 (0.93–1.33)	0.172	1.02 (0.98–1.06)
Model 2	Reference	0.95 (0.81–1.11)	1.00 (0.85–1.17)	1.01 (0.85–1.20)	0.743	0.99 (0.94–1.05)
Model 3	Reference	0.94 (0.81–1.10)	1.00 (0.85–1.18)	1.02 (0.85–1.21)	0.683	0.99 (0.94–1.04)
mRS score 3–6						
*n* (%)	374 (11.09)	420 (11.63)	426 (12.71)	576 (16.31)		
Unadjusted	Reference	1.06 (0.89–1.25)	1.17 (0.99–1.38)	1.56 (1.33–1.83)	<0.001	1.12 (1.03–1.21)
Model 1	Reference	1.10 (0.92–1.31)	1.25 (1.06–1.47)	1.81 (1.54–2.12)	<0.001	1.16 (1.03–1.30)
Model 2	Reference	1.02 (0.84–1.23)	1.15 (0.97–1.38)	1.47 (1.24–1.75)	<0.001	1.08 (1.00–1.17)
Model 3	Reference	1.01 (0.83–1.22)	1.15 (0.96–1.37)	1.47 (1.22–1.76)	<0.001	1.08 (1.00–1.17)

*Note*: Hazard ratios (HRs) with 95% confidence intervals (CIs) were used for death and stroke recurrence; Odds ratios (ORs) with 95% CIs were used for mRS scores 3–6. Model 1: adjusted for age and sex. Model 2: adjusted for age, sex, body mass index, current smoker, current alcohol drinking, disease history (hypertension, stroke or transient ischemic attacks, diabetes, dyslipidemia, atrial fibrillation, arthritis), the National Institutes of Health Stroke Scale score at admission, stroke subtype, prestroke modified Rankin Scale score, stroke etiology, antihypertensive agents, cholesterol‐lowering agents, and hypoglycemic agents. Model 3: adjusted for variables in model 2, plus total cholesterol, triglyceride, and low‐density lipoprotein.

Abbreviations: MHR indicates monocyte‐to‐HDL ratio; mRS, modified Rankin Scale; SD, standard deviation.

Figures S1 and S2 present the associations between per 1 SD increase in MHR and clinical outcomes. All of these analyses were adjusted for age, sex, BMI, current smoker, current alcohol drinking, disease history (hypertension, stroke or TIAs, diabetes, dyslipidemia, atrial fibrillation, and arthritis), the NIHSS score at admission, stroke subtype, prestroke mRS score, stroke etiology, antihypertensive agents, cholesterol‐lowering agents, hypoglycemic agents, TC, TG, and LDL except for the covariate that was stratified. The associations were almost consistent with respect to BMI, and history of stroke or TIA (*p* for interactions > 0.05). Significant heterogeneity was observed across age groups for the associations between MHR and clinical outcomes (except for death within 3 months); these associations were more pronounced among younger patients (*p* for interactions < 0.05). Significant interactions between MHR and stroke etiology group for mRS of 3–6 or 2–6 at 1 year were detected (*p* for interactions < 0.05); the positive correlations seem more pronounced among large artery atherosclerosis (LAA) and cardiogenic embolism patients.

### 3.3 Incremental predictive value of MHR

We evaluated the performance of models with MHR to predict all‐cause death, stroke recurrence, and poor functional outcome (Table 3). For all‐cause death within 1 year, the *C*‐statistic by the conventional model significantly improved with the addition of MHR (from 0.804 to 0.809; *p* = 0.010). The MHR also improved predictive performance validated by category‐free NRI (17.73%; 95% CI, 8.50–26.96; *p* < 0.001). However, the discriminatory power appeared to be substantially indistinguishable (IDI, 0.153%, *p* = 0.182). No significant improvements were observed after the addition of MHR to the basic model for the prediction of stroke recurrence within 1 year (*C*‐statistic, IDI, and NRI, all *p* > 0.05). For poor functional outcome (mRS 3–6) at 1 year, the addition of MHR to the basic model improved the predictive performance according to the *C*‐statistic (from 0.804 to 0.807; *p* = 0.001), IDI (0.242%; 95%CI, 0.110–0.370; *p* < 0.001), and category‐free NRI (15.63%; 95% CI, 10.74–20.51; *p* < 0.001), respectively. Similar results were found for outcomes at 3 months. The predictive performance of the addition of MHR to the basic model for poor functional outcome defined as mRS of 2–6 is presented in Table S3.

**TABLE 3:** Performance of models with MHR to predict all‐cause death, stroke recurrence and poor functional outcome.

Model	*C*‐statistic		IDI		Category‐free NRI^a^	
	Estimate (95% CI)	*p* Value	Estimate (95% CI), %	*p* Value	Estimate (95% CI), %	*p* Value
At 3 months						
Death						
Basic model^b^	0.837 (0.807–0.866)	Reference	Reference		Reference	
Basic model + MHR	0.844 (0.817–0.872)	0.050	0.361 (−0.110, 0.830)	0.129	25.29 (11.66, 38.92)	<0.001
Stroke recurrence						
Basic model^b^	0.639 (0.620–0.658)	Reference	Reference		Reference	
Basic model + MHR	0.640 (0.621–0.658)	0.583	0.016 (−0.010–0.040)	0.164	4.40 (−2.58–11.38)	0.217
mRS score 3–6						
Basic model^b^	0.817 (0.807–0.828)	Reference	Reference		Reference	
Basic model + MHR	0.819 (0.808–0.829)	0.084	0.140 (0.050–0.230)	0.004	10.51 (5.89–15.13)	<0.001
At 1 year						
Death						
Basic model^b^	0.804 (0.783–0.826)	Reference	Reference		Reference	
Basic model + MHR	0.809 (0.788–0.831)	0.010	0.153 (−0.070, 0.380)	0.182	17.73 (8.50, 26.96)	<0.001
Stroke recurrence						
Basic model^b^	0.623 (0.607–0.638)	Reference	Reference		Reference	
Basic model + MHR	0.623 (0.607–0.639)	0.581	0.009 (−0.010–0.030)	0.259	2.81 (−2.11–7.72)	0.333
mRS score 3–6						
Basic model^b^	0.804 (0.793–0.815)	Reference	Reference		Reference	
Basic model + MHR	0.807 (0.796–0.818)	0.001	0.242 (0.110–0.370)	<0.001	15.63 (10.74, 20.51)	<0.001

Abbreviations: CI, confidence interval; IDI, integrated discrimination improvement; MHR, monocyte‐to‐HDL ratio; mRS, modified Rankin Scale; NRI, net reclassification index.

^a^ Patients were divided into three risk categories: 0%–5%, 5%–20%, and 20%–100%.

^b^ Basic model included adjusted for age, sex, body mass index, current smoker, current alcohol drinking, disease history (hypertension, stroke or transient ischemic attacks, diabetes, dyslipidemia, atrial fibrillation, arthritis), the National Institutes of Health Stroke Scale score at admission, stroke subtype, prestroke modified Rankin Scale score, stroke etiology, antihypertensive agents, cholesterol‐lowering agents, hypoglycemic agents, total cholesterol, triglyceride, and low‐density lipoprotein.

## 4 DISCUSSION

In this prospective study, we found that a higher level of MHR was independently associated with the increased risk of poor functional outcome and all‐cause death, in patients with ischemic stroke or TIA at 3 months and 1‐year follow‐ups. Although MHR was not related to stroke recurrence within 3 months and 1 year, it was still considered a useful predictor for short‐ or long‐term prognosis among ischemic stroke or TIA patients. Collectively, our findings highlight the clinical relevance of evaluating MHR value in patients with ischemic stroke or TIA at admission.

Prior studies reported that MHR may be considered a novel marker for cardiovascular diseases and a predictor of prognosis in patients with inflammatory diseases. A cross‐sectional population‐based study analyzed 8148 individuals and demonstrated a linear relation between MHR and ischemic stroke.^17^ Results from the National Health and Nutrition Examination Survey suggested MHR value was independently related to all‐cause and cardiovascular mortality in the general population.^25^ Another prospective observational study suggested that MHR was an independent predictor of major adverse cardiovascular events' occurrence in patients undergoing coronary angiography.^16^ Recently, a meta‐analysis that involved 9 studies indicated that for patients with coronary heart disease, increased MHR value might be associated with higher long‐term mortality and major adverse cardiovascular events.^26^ Nevertheless, some studies have explored the relationship between MHR and short‐term stroke prognosis based on small sample size data from one single center but reported inconsistent results for hemorrhagic transformation^27^, ^28^ and for all‐cause mortality.^20^, ^22^ The discrepancy might be due to the differences in the patients' characteristics, and the small sample size with limited statistical power. Furthermore, evidence surrounding the relationship between MHR and the long‐term prognosis of stroke was limited. Our study confirmed the correlations between MHR and short‐ and long‐term poor functional outcomes and all‐cause death in ischemic stroke or TIA patients, especially among younger or LAA and cardiogenic embolism patients, in accordance with the previously reported relationship between higher levels of MHR and poor functional outcome at 3 months.^29^, ^30^ In addition, we further proved the predictive value of MHR for poor functional outcome and all‐cause death after stroke using comprehensive evaluation indicators including C statistics, IDI, and NRI. To the best of our knowledge, no previous study investigated the relationship between MHR and post‐stroke recurrence. Our study showed a null association between MHR and recurrent stroke in patients with a minor ischemic stroke or TIA; however, further research is warranted to validate this result.

Atherosclerosis is a highly prevalent cause of ischemic stroke,^31^ due to associated inflammation and lipid accumulation.^1^, ^2^ Monocytes are an important source of pro‐inflammatory substances during atherosclerosis,^8^, ^9^, ^32^ and have found to be obviously associated with elevated risks of death and poor functional outcomes at 3‐months and 1‐year follow‐up in patients with acute ischemic cerebrovascular events.^33^ In the onset of AIS, monocytes bind to adhesion molecules on injured vascular endothelium, migrate to the sub‐endothelial space, and mature into macrophages, then phagocytose oxidized LDL and differentiate into foam cells.^34^ Foam cells can release a variety of pro‐inflammatory and pro‐oxidative cytokines at the inflammatory site, attracting T lymphocytes and more monocytes to gather.^35^ Monocytes directly or indirectly participate in all stages of atherosclerosis from fat streak formation to plaque rupture.^22^ HDL has anti‐inflammatory, antioxidant, and antithrombotic effects, and is a classic anti‐atherosclerotic factor.^36^, ^37^ A prospective cohort study found that changes in HDL proteins during the early acute phase of stroke was associated with recovery.^38^ Another study enrolled AIS patients with diabetes reported that HDL levels were negatively correlated with the risk of recurrent stroke and major adverse cardiovascular events within 1 year.^39^ HDL has also been found to be an independent risk factor for 3‐month poor outcomes in patients with ischemic stroke.^40^ This may be related to the fact that HDL can prevent monocytes from recruiting to the arterial wall, thereby inhibiting the adhesion of monocytes to endothelial cells, reducing the activation of monocytes and the proliferation of monocyte progenitor cells,^41^ thereby protecting endothelial cells from inflammation and oxidative stress.^19^ Therefore, we speculate that when monocyte levels increase and/or HDL levels decrease, patients with ischemic stroke would be more adversely affected by inflammation and lipid accumulation, and the degree of atherosclerosis could be more severe. That is, high MHR levels are more likely to lead to irreversible damage to brain tissue, which in turn affects all‐cause death and poor functional outcomes in ischemic stroke. This is consistent with our analyses of subgroups of stroke subtypes. Furthermore, younger, LAA, and cardioembolism patients were more likely to have higher MHR levels in our study. The associations between MHR and clinical outcomes were more pronounced among younger patients after multivariable adjustment for important potential confounders. This may be related to the following mechanism. Hearps et al. found that the expression of CD11b, L‐selectin, and TLR4 in the elderly may affect the migration of monocytes and lead to more dysfunction of monocytes than in young people.^42^ In addition, it was found that the level of P38 in older mice was significantly lower than that in young mice in an animal study.^43^ HDL further restrains monocytes by inhibiting the expression of P38 and CD11b.^41^, ^44^ Therefore, eld may be a protective factor. In addition to LAA, we also obtained similar results among the cardioembolism patients, which are different from previous studies.^29^, ^30^ We speculate that the degree of correlation between MHR levels and 1‐year poor functional prognosis in cardioembolism is related to the inflammatory potential of monocytes or HDL. Monocytes adhered to the surface of endothelial cells can induce the production of various cytokines in damaged tissues and stimulate the proliferation of fibroblasts.^45^ Extensive proliferation of fibroblasts can increase atrial fibrosis, alter atrial conduction characteristics,^46^ induce atrial fibrillation, and cause shedding of cardiac embolus. However, HDL can have a protective effect on atrial muscle cells by increasing the directional transport of cholesterol.^19^ The interaction between inflammation and lipids can not only promote the occurrence and development of atherosclerosis but also drive the healing response of vascular injury, which is not easy to cause stroke recurrence and may play a protective role, so we have not found a correlation between high levels of MHR and stroke recurrence.^1^, ^2^, ^47^ Recently, with the continuous progress of gene‐level research, the differences in the transcriptional process of monocytes after ischemic stroke may be another potential reason for the different tendencies of different subtypes of stroke.^48^ In addition, recent studies reported some novel prognostic biomarkers for predicting stroke outcomes, such as circulating immunoregulatory lymphocytes,^49^ neurofilament light chain protein,^50^ and circulating microRNA.^51^ However, we could not adjust them due to the lack of data. Therefore, we cannot conclude the casual relationship.

The strengths of our study include the multicenter prospective registry design and large sample size. Nevertheless, there were still some limitations to our study. First, this study only captured baseline MHR and did not measure blood monocyte count and HDL concentration at discharge or during follow‐up. The exploration of the dynamic change in MHR might provide more valuable information about the mechanism. Our study cannot provide strong evidence that MHR might be a new therapeutic target to improve the prognosis of stroke patients compared to previous studies. Second, equipment heterogeneity at each participating center may lead to biased estimates, but this may have little impact since all the equipment has strict quality control in daily use. Thirdly, although we have adjusted multiple potential covariates including cholesterol‐lowering agents, dietary^52^ (niacin, legumes, nuts, olive oil, etc.) and other pharmacological interventions^53^ (such as cholesterylester transfer protein inhibitors) the patients received after admission may affect HDL levels and results. Due to the lack of this information, residual confounding factors could not be excluded. We cannot establish a causal relationship between MHR with clinical outcomes after stroke. Finally, HDL‐specific genotypes or phenotypes (e.g., familial hypercholesterolemia or hyperalphalipoproteinemia) were not screened in our study, our results may not be generalizable to a more heterogeneous global population.

## 5 CONCLUSIONS

In conclusion, our study suggested that elevated MHR levels at admission substantially increased risks of all‐cause death and poor functional outcomes in patients with ischemic stroke or TIA at 3‐month and 1‐year follow‐ups. The finding underscored the utility and predictive potential of MHR, which can be swiftly applied clinically to determine the prognosis of stroke.

## AUTHOR CONTRIBUTIONS

Q.X. and Q.W. conducted the literature review, interpreted the data, and drafted the initial manuscript; Q.X. performed the statistical analyses; L.C., interpreted data, reviewed, and revised the manuscript. X.M. and A.W. designed the study and were involved in data interpretation and manuscript preparation; H.L., X.T., X.X., Y.Z., X.Z, and Y.J.W. contributed to the acquisition of data; Y.L., Y.P.W., and Y.J.W. revised the manuscript; and all authors have read and approved the submitted manuscript.

## FUNDING INFORMATION

This work was supported by National Key Research and Development Program of China (2022YFC3600600 and 2022YFC2502400), National Natural Science Foundation of China (81870905, U20A20358, and 82111530203), Training Fund for Open Projects at Clinical Institutes and Departments of Capital Medical University (CCMU2022ZKYXZ009), Chinese Academy of Medical Sciences Innovation Fund for Medical Sciences (2019‐I2M‐5‐029), and the Capital's Funds for Health Improvement and Research (2020‐1‐2041).

## CONFLICT OF INTEREST STATEMENT

The authors declare that there is no conflict of interest.

## Supporting information


Appendix S1

## Data Availability

Data are available to researchers on request for purposes of reproducing the results or replicating the procedure by directly contacting the corresponding author.
