# Supplementary material for: Monocyte to high‐density lipoprotein ratio predicts clinical outcomes after acute ischemic stroke or transient ischemic attack
Source: CNS Neurosci Ther. 2023 Mar 13;29(7):1953–64. doi: 10.1111/cns.14152 (PMC10324347; doi:10.1111/cns.14152)
Supplement: Supplementary file 1 — Appendix S1 [file CNS-29-1953-s001.docx]

**Supplemental Materials for**

**Monocyte to High-Density Lipoprotein Ratio Predicts Clinical Outcomes after Acute Ischemic Stroke or Transient Ischemic Attack**

**Running title: MHR and outcomes after stroke**

**Table S1. Baseline characteristics between included and excluded patients**

| **Characteristics** | **Total** | **Exclude** | **Include** | ***P*** |
| --- | --- | --- | --- | --- |
| No. of the patients | 15166 | 1301 | 13865 |  |
| Age, median (IQR), y | 63 (54-70) | 63 (54-71) | 62 (54-70) | 0.037 |
| Women, n (%) | 4802 (31.66) | 367 (28.21) | 4435 (31.99) | 0.005 |
| BMI, median (IQR), kg/m^2^ | 24.49 (22.6-26.56) | 24.49 (22.67-26.56) | 24.49 (22.6-26.56) | 0.891 |
| Current smoker, n (%) | 4752 (31.33) | 450 (34.59) | 4302 (31.03) | 0.008 |
| Current alcohol drinking, n (%) | 6797 (44.82) | 583 (44.81) | 6214 (44.82) | 0.997 |
| **Medical History, n (%)** |  |  |  |  |
| Hypertension | 9494 (62.6) | 828 (63.64) | 8666 (62.50) | 0.416 |
| Stroke or TIA | 3675 (24.23) | 299 (22.98) | 3376 (24.35) | 0.271 |
| Diabetes | 3510 (23.14) | 287 (22.06) | 3223 (23.25) | 0.332 |
| Dyslipidemia | 1191 (7.85) | 95 (7.30) | 1096 (7.90) | 0.439 |
| Atrial fibrillation | 1019 (6.72) | 95 (7.30) | 924 (6.66) | 0.379 |
| Coronary heart disease | 1608 (10.6) | 131 (10.07) | 1477 (10.65) | 0.513 |
| Heart failure | 94 (0.62) | 10 (0.77) | 84 (0.61) | 0.474 |
| Peripheral vascular disease | 118 (0.78) | 7 (0.54) | 111 (0.80) | 0.303 |
| Arthritis | 329 (2.17) | 26 (2.00) | 303 (2.19) | 0.658 |
| **Admission stroke data** |  |  |  |  |
| NIHSS at admission, median (IQR) | 3 (1-6) | 3 (2-6) | 3 (1-6) | <0.001 |
| Prestroke mRS score 2-5, n (%) | 1344 (8.86) | 117 (8.99) | 1227 (8.85) | 0.862 |
| Stroke subtype, n (%) |  |  |  | 0.005 |
| Ischemic stroke | 14146 (93.27) | 1238 (95.16) | 12908 (93.10) |  |
| TIA | 1020 (6.73) | 63 (4.84) | 957 (6.90) |  |
| Stroke etiology, n (%) |  |  |  | 0.185 |
| Large-artery atherosclerosis | 3856 (25.43) | 364 (27.98) | 3492 (25.19) |  |
| Cardioembolism | 917 (6.05) | 73 (5.61) | 844 (6.09) |  |
| Small-vessel occlusion | 3165 (20.87) | 249 (19.14) | 2916 (21.03) |  |
| Other determined etiology | 182 (1.2) | 15 (1.15) | 167 (1.20) |  |
| Undetermined etiology | 7046 (46.46) | 600 (46.12) | 6446 (46.49) |  |
| **Laboratory data, median (IQR)** |  |  |  |  |
| TC, mmol/L | 4.14 (3.43-4.9) | 4.04 (3.34-4.73) | 4.14 (3.44-4.91) | 0.002 |
| TG, mmol/L | 1.37 (1.02-1.91) | 1.35 (1.03-1.79) | 1.37 (1.02-1.92) | 0.087 |
| LDL, mmol/L | 2.44 (1.84-3.11) | 2.34 (1.77-2.97) | 2.45 (1.85-3.11) | 0.003 |
| HDL, mmol/L | 1.08 (0.91-1.29) | 1.05 (0.87-1.24) | 1.09 (0.91-1.3) | <0.001 |
| Monocyte, 10^9^/L | 0.42 (0.32-0.54) | 0.42 (0.33-0.58) | 0.42 (0.32-0.54) | 0.009 |
| MHR | 0.39 (0.27-0.53) | 0.41 (0.29-0.57) | 0.39 (0.27-0.53) | 0.013 |
| **Treatment in hospital, n (%)** |  |  |  |  |
| Antihypertensive agents | 7000 (46.5) | 616 (48.13) | 6384 (46.35) | 0.224 |
| Antiplatelet agents | 14613 (97.08) | 1226 (95.78) | 13387 (97.20) | 0.004 |
| Anticoagulant agents | 1546 (10.27) | 155 (12.11) | 1391 (10.10) | 0.023 |
| Cholesterol-lowering agents | 14506 (96.37) | 1236 (96.56) | 13270 (96.35) | 0.695 |
| Hypoglycemic agents | 3792 (25.19) | 315 (24.61) | 3477 (25.25) | 0.616 |
| rt-PA intravenous thrombolytic | 1303 (8.59) | 135 (10.38) | 1168 (8.42) | 0.016 |
| Mechanical thrombectomy | 39 (0.26) | 0 (0.00) | 39 (0.28) | 0.055 |

BMI indicates body mass index; HDL, high-density lipoprotein; IQR, interquartile range; LDL, low-density lipoprotein; MHR, monocyte‑to‑HDL ratio; mRS, modified Rankin Scale; NIHSS, the National Institutes of Health Stroke Scale; TC, total cholesterol; TG, triglyceride; TIA, transient ischemic attack.

**Table S2. Associations of MHR with poor functional outcome defined as mRS of 2-6**

| **Outcomes** | **Quartiles of MHR** | | | |  | **Per 1 SD increase** |
| --- | --- | --- | --- | --- | --- | --- |
|  | **Q1** | **Q2** | **Q3** | **Q4** | ***P* for trend** |  |
| **mRS score 2-6 at 3 months** |  |  |  |  |  |  |
| n (%) | 816 (24.21) | 861 (23.85) | 835 (24.90) | 1023 (28.97) |  |  |
| Unadjusted | Reference | 0.98(0.87-1.11) | 1.04(0.89-1.21) | 1.28(1.10-1.48) | 0.001 | 1.10(1.02-1.19) |
| Model1 | Reference | 1.02(0.90-1.15) | 1.10(0.94-1.30) | 1.43(1.22-1.68) | <0.001 | 1.14(1.03-1.27) |
| Molde2 | Reference | 0.95(0.83-1.08) | 1.01(0.86-1.20) | 1.18(0.99-1.40) | 0.043 | 1.06(0.99-1.14) |
| Model3 | Reference | 0.97(0.85-1.11) | 1.05(0.88-1.24) | 1.24(1.04-1.49) | 0.012 | 1.07(0.99-1.16) |
| **mRS score 2-6 at 1 year** |  |  |  |  |  |  |
| n (%) | 720 (21.36) | 774 (21.44) | 760 (22.67) | 968 (27.41) |  |  |
| Unadjusted | Reference | 1.00(0.89-1.13) | 1.08(0.93-1.25) | 1.39(1.21-1.60) | <0.001 | 1.11(1.03-1.20) |
| Model1 | Reference | 1.04(0.92-1.18) | 1.14(0.99-1.33) | 1.57(1.36-1.81) | <0.001 | 1.16(1.04-1.28) |
| Molde2 | Reference | 0.98(0.85-1.12) | 1.06(0.91-1.24) | 1.31(1.12-1.53) | <0.001 | 1.07(1.00-1.16) |
| Model3 | Reference | 0.97(0.85-1.11) | 1.06(0.91-1.24) | 1.32(1.13-1.54) | <0.001 | 1.08(0.99-1.17) |

Hazard ratios (HRs) with 95% confidence intervals (CIs) were used for death and stroke recurrence; Odds ratios (ORs) with 95% CIs were used for mRS score 3-6.

MHR indicates monocyte‑to‑HDLratio; mRS, modified Rankin Scale; SD, standard deviation.

Model 1: adjusted for age and sex.

Model 2: adjusted for age, sex, body mass index, current smoker, current alcohol drinking, disease history (hypertension, stroke or transient ischemic attacks, diabetes, dyslipidemia, atrial fibrillation, arthritis), the National Institutes of Health Stroke Scale score at admission, stroke subtype, prestroke modified Rankin Scale score, stroke etiology, antihypertensive agents, cholesterol-lowering agents, and hypoglycemic agents.

Model 3: adjusted for variables in model 2, plus total cholesterol, triglyceride, and low-density lipoprotein.

**Table S3. Performance of models with MHR to predict poor functional outcome defined as mRS of 2-6**

| **Model** | **C-statistic** | |  | **IDI** | |  | **Category-free NRI^*^** | |
| --- | --- | --- | --- | --- | --- | --- | --- | --- |
|  | **Estimate (95% CI)** | ***P* value** |  | **Estimate (95% CI), %** | ***P* value** |  | **Estimate (95% CI), %** | ***P* value** |
| **mRS score 2-6 at 3 months** |  |  |  |  |  |  |  |  |
| Basic model^†^ | 0.793 (0.784-0.801) | Reference |  | Reference |  |  | Reference |  |
| Basic model+MHR | 0.794 (0.785-0.802) | 0.021 |  | 0.109 (0.040-0.180) | 0.001 |  | 9.44 (5.78, 13.10) | <0.001 |
| **mRS score 2-6 at 1 year** |  |  |  |  |  |  |  |  |
| Basic model^†^ | 0.769 (0.759-0.778) | Reference |  | Reference |  |  | Reference |  |
| Basic model+MHR | 0.771 (0.761-0.780) | 0.002 |  | 0.179 (0.090, 0.270) | <0.001 |  | 11.86 (8.11, 15.61) | <0.001 |

CI indicates confidence interval; IDI, integrated discrimination improvement; MHR, monocyte‑to‑HDL ratio; mRS, modified Rankin Scale; NRI, net reclassification index.

^*^Patients were divided into 3 risk categories: 0% to 5%, 5% to 20%, and 20% to 100%.

^†^Basic model included adjusted for age, sex, body mass index, current smoker, current alcohol drinking, disease history (hypertension, stroke or transient ischemic attacks, diabetes, dyslipidemia, atrial fibrillation, arthritis), the National Institutes of Health Stroke Scale score at admission, stroke subtype, prestroke modified Rankin Scale score, stroke etiology, antihypertensive agents, cholesterol-lowering agents, hypoglycemic agents, total cholesterol, triglyceride, and low-density lipoprotein.


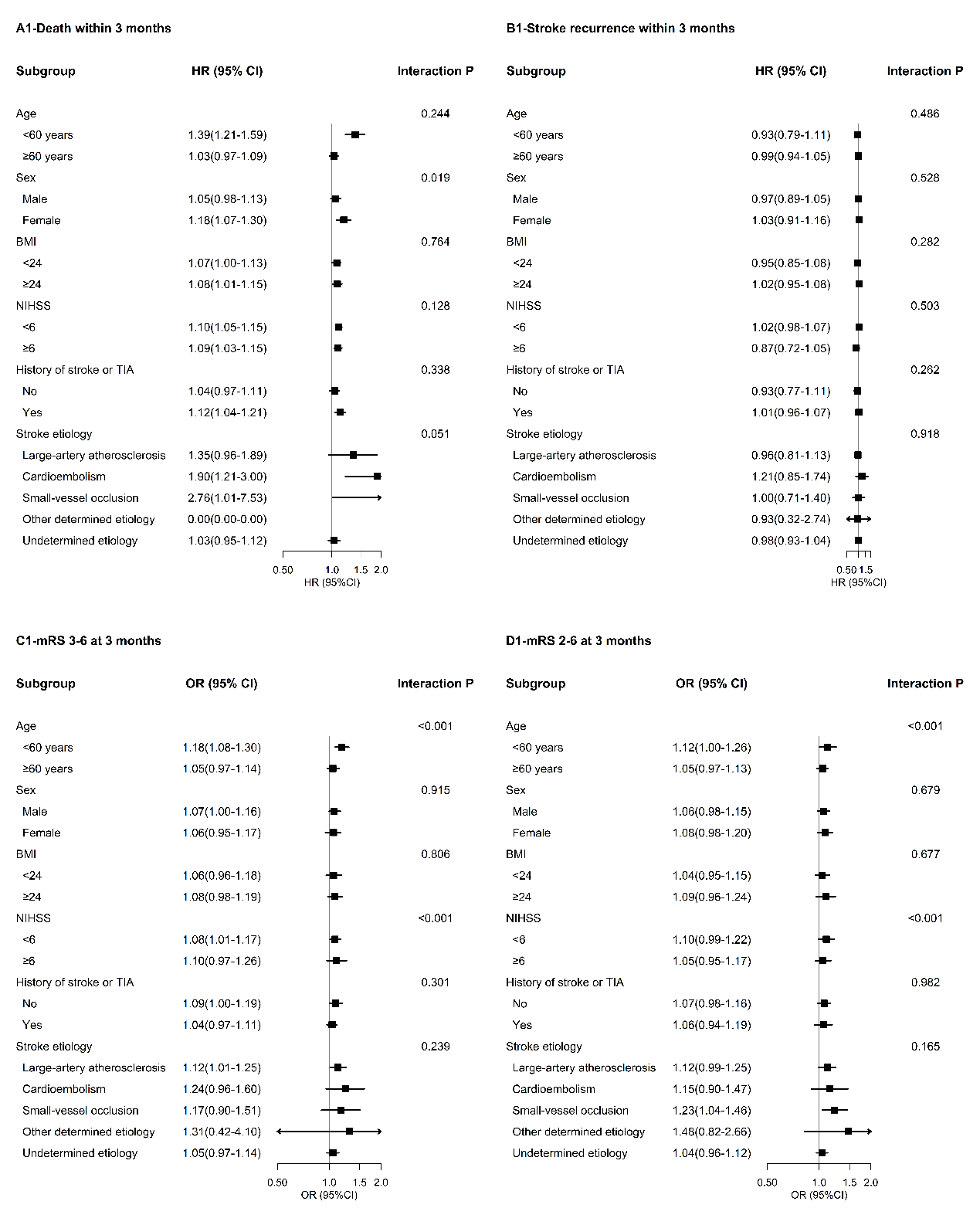


**Figure S1. Subgroup analysis of association between per 1 standard deviation MHR increase and clinical outcomes during 3 months follow-up.**

Adjusted for age, sex, body mass index, current smoker, current alcohol drinking, disease history (hypertension, stroke or transient ischemic attacks, diabetes, dyslipidemia, atrial fibrillation, arthritis), the National Institutes of Health Stroke Scale score at admission, stroke subtype, prestroke modified Rankin Scale score, stroke etiology, antihypertensive agents, cholesterol-lowering agents, hypoglycemic agents, total cholesterol, triglyceride, and low-density lipoprotein except for the covariate that was stratified.

**
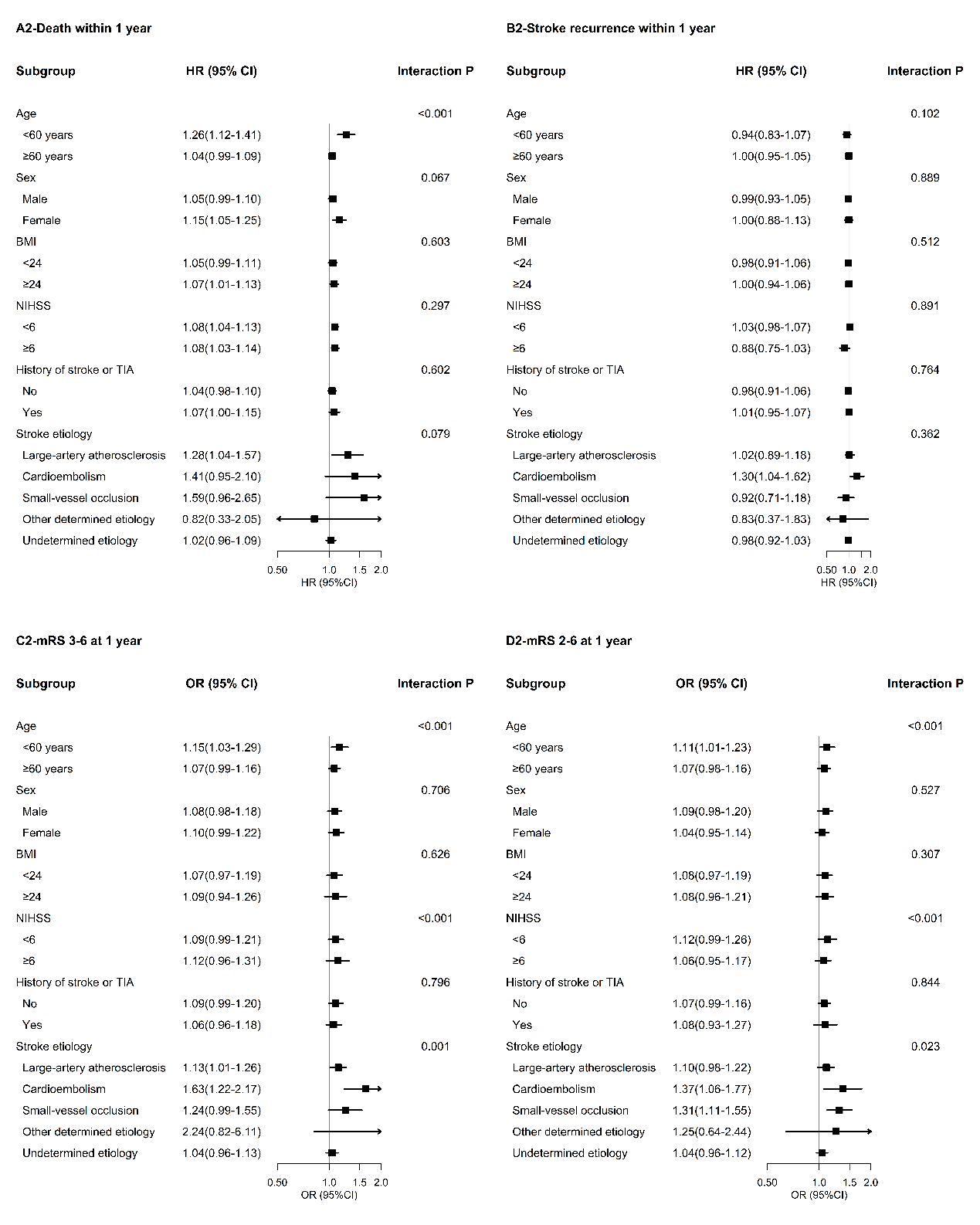
**

**Figure S2. Subgroup analysis of association between per 1 standard deviation MHR increase and clinical outcomes during 1 year follow-up.**

Adjusted for age, sex, body mass index, current smoker, current alcohol drinking, disease history (hypertension, stroke or transient ischemic attacks, diabetes, dyslipidemia, atrial fibrillation, arthritis), the National Institutes of Health Stroke Scale score at admission, stroke subtype, prestroke modified Rankin Scale score, stroke etiology, antihypertensive agents, cholesterol-lowering agents, hypoglycemic agents, total cholesterol, triglyceride, and low-density lipoprotein except for the covariate that was stratified.
